# Primary Kaposi sarcoma of the glans: A rare case in an HIV‐negative patient

**DOI:** 10.1002/ccr3.2838

**Published:** 2020-04-13

**Authors:** Farnaz Araghi, Mohammadreza Tabary, Farahnaz Bidari‐Zerehpoosh, Zahra Asadi‐Kani, Reza M. Robati

**Affiliations:** ^1^ Skin Research Center Shahid Beheshti University of Medical Sciences Tehran Iran; ^2^ School of Medicine Tehran University of Medical Sciences Tehran Iran; ^3^ Department of Pathology Loghman Hakim Hospital Shahid Beheshti University of Medical Sciences Tehran Iran; ^4^ Department of Dermatology Loghman Hakim Hospital Shahid Beheshti University of Medical Sciences Tehran Iran

**Keywords:** genitalia, glans penis, HHV‐8, Kaposi sarcoma

## Abstract

First presentation of the Kaposi sarcoma (KS) on the penis is not prevalent, and it was reported in 2%‐3% of the cases that mostly occurred in the HIV‐positive patients. Here, we report a case of primary KS on the glans penis in an HIV‐negative patient.

## Graphical abstract

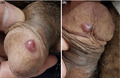

## 1 INTRODUCTION

Kaposi sarcoma (KS) was first introduced by Moris Kaposi, a Hungarian dermatologist, in 1872.^1^ The association of Kaposi sarcoma and the human herpesvirus 8 (HHV‐8) was then studied in 1994. Kaposi sarcoma is defined as a multifocal angioproliferative disorder that originates from endothelial cells. Generally, KS may present as a red, violet, or black nodule especially on the skin, respiratory, and gastrointestinal tract.^1^ Further, KS mainly affects mucocutaneous sites rather than visceral organs.^2^ KS is more common in men.^3^ As a systemic disease, KS has several forms that have been reported in previous studies. Classical form is dominant in adults and occurs mostly in the Mediterranean region, where HHV‐8 is more common. The most aggressive form is the endemic one that is mainly observed in HIV and immune‐compromised patients.

First presentation of the KS on the penis is not prevalent, and it was reported in 2%‐3% of the cases that mostly occurred in patients with AIDS.^4^ Therefore, the report of the primary non–HIV‐related KS in the penis is rare in the literature. Here, we report a patient with a classic form of KS who had a primary penile lesion.

## 2 CASE PRESENTATION

A 50‐year‐old man was admitted to our dermatology clinic at our teaching hospital. He presented with erythematous nodular penile lesions of 4 months' duration. There was no history of fever, weight loss, or any other symptoms. He also denied any history of systemic disease. Our patient was married with no history of extramarital relationship.

**Figure 1:**
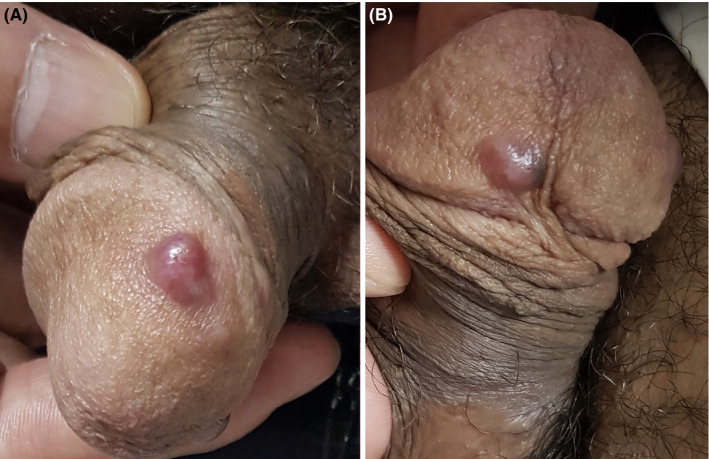
A, a reddish nodule on the dorsal side of glans penis; B, a reddish nodule on the ventral side of the glans penis

In his physical examination, there were some reddish, nontender nodules of 5‐10mm on the ventral and dorsal sides of glans penis (Figure 1). No other skin lesion was observed on his skin. There were no palpable lymph nodes in the inguinal region.

**Figure 2:**
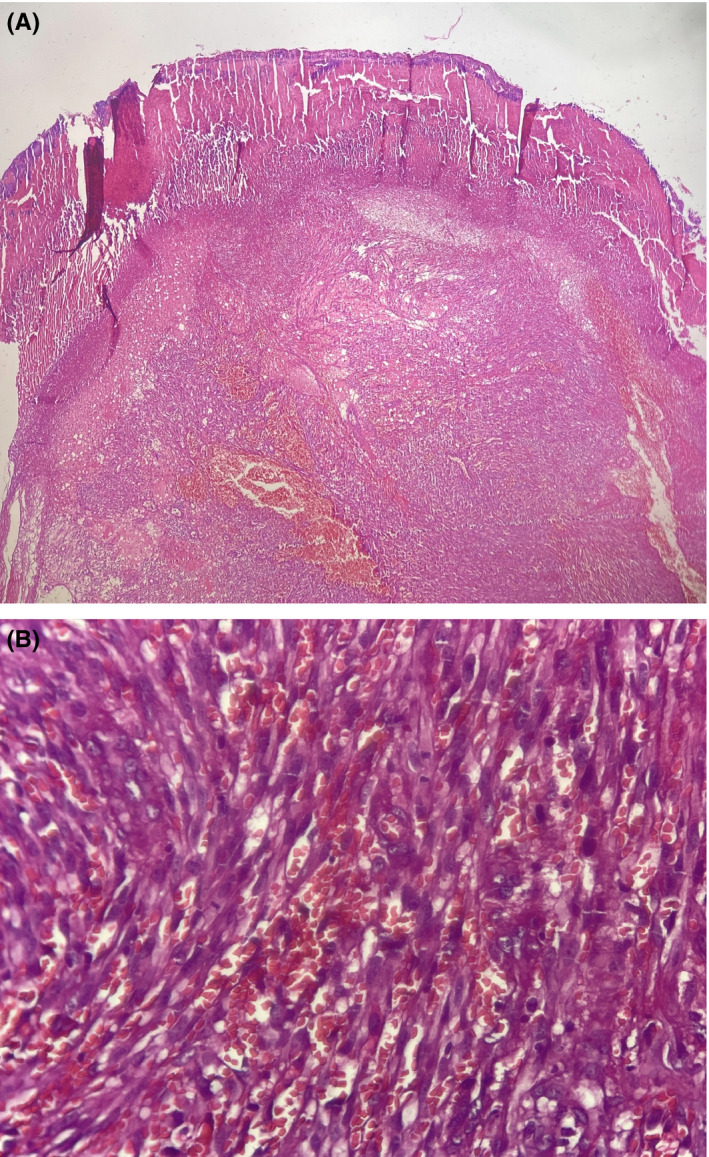
A, a nodular lesion with focal ulceration (H&E ×4); B, Atypical spindle cell proliferation, forming slit‐like vessels grouped in bundles and RBC extravasation (H&E ×40)

**Figure 3:**
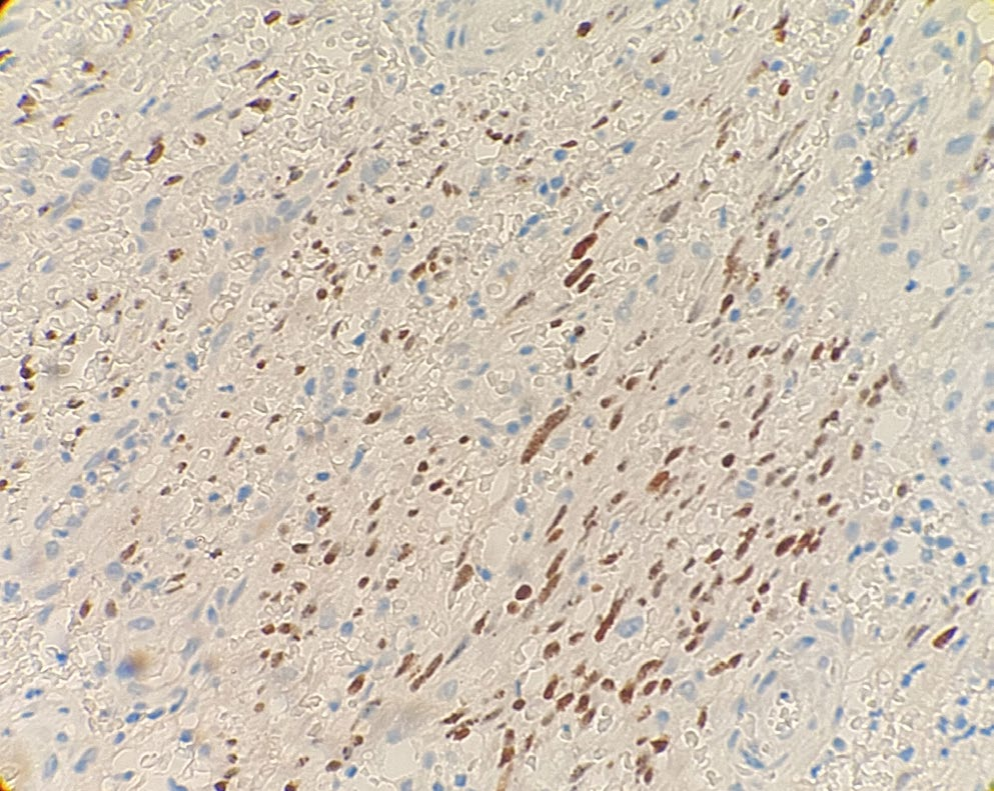
Immunohistochemistry assay confirmed Kaposi sarcoma associated with HHV‐8, strongly positive for LNA‐1

Then skin biopsy was taken from the lesion, and the histopathology examination revealed atypical spindle cell proliferation, forming slit‐like vessels grouped in bundles and RBC extravasation (Figure 2). Furthermore, the immunohistochemical study was performed and the pathology confirmed the diagnosis of KS based on a positive HHV‐8 immunostaining (Figure 3).

**Figure 4:**
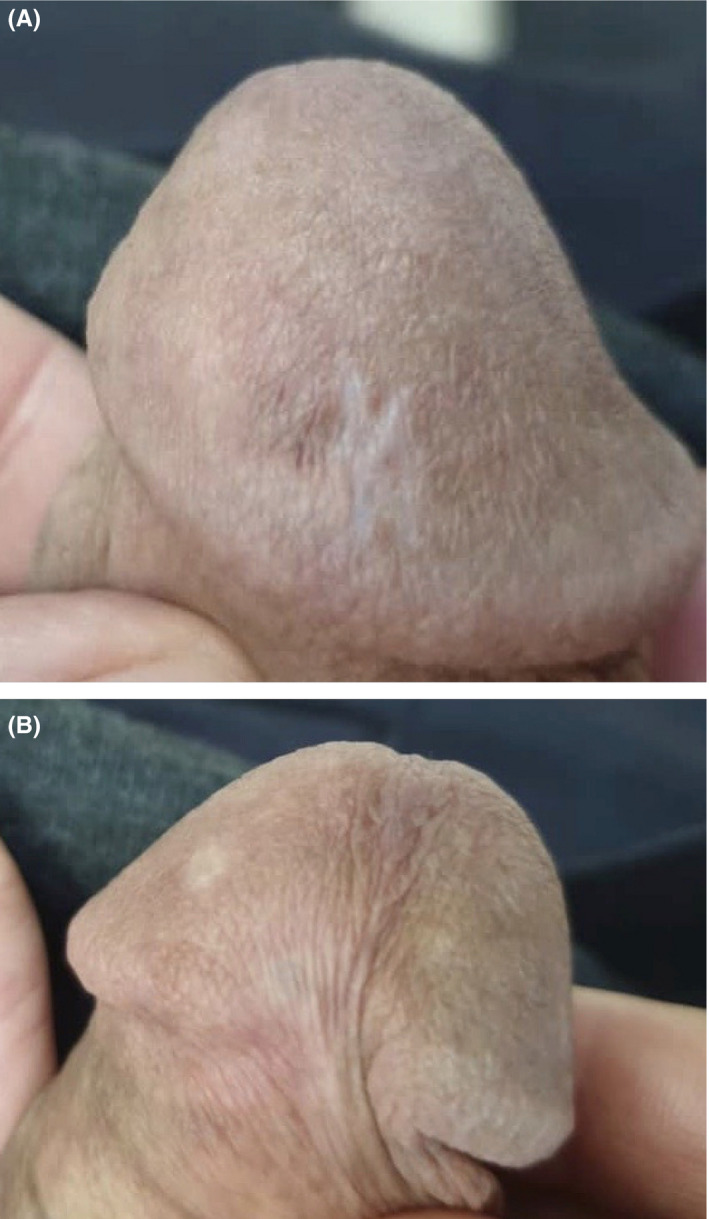
Complete resolution of the lesion after performing cryotherapy on A. dorsal side of glans penis and B. ventral side of glans penis

All of his laboratory tests including HIV viral serology were negative. His chest X‐ray was normal. No enlarged lymph nodes were found in his abdominopelvic ultrasonography. In addition, the patient was evaluated for the presence of any other systemic involvement and the result was negative. Our patient underwent cryotherapy for 3 courses with complete disappearance of penile lesions, and no local recurrence occurred after one year (Figure 4).

## 3 DISCUSSION

As a malignant tumor, KS originates from lymphatic endothelial cells. Classic, endemic, epidemic, iatrogenic, and nonepidemic forms are the 5 types of KS.^5^

Although genital lesions were observed in 20% of KS cases, only 3% of them had the primary localized lesion on the glans penis.^6^ The most prevalent form of KS is the nodular form which is found in 83% of the cases.^7^ The rare classic type that mostly occurs in the Mediterranean and Eastern Europe population may be also observed in the lower extremities of elderly non‐HIV patients between the 6th and 7th decades of age.^8^ The infrequent presentation of this classic type is primary penile lesions similar to our patient, which is considered mostly to associate with HHV‐8 as an indispensable factor. Table 1 summarizes some relevant cases of genital KS and other associated features according to their publication date.

**Table 1:** Similar Cases of Genital Kaposi Sarcoma and the Associated Features

Sex/Age	Location	Presentation	Underlying condition	HHV‐8 association	Treatment	First author's name, reference number/Y
Female/33 y	Vulvar region	Persistent nodular region	HIV & syphilis coinfection	Not mentioned	Not mentioned	dos Reis HL^10^/2019
Male/63 y	Glans penis	Ulcerative nodule	Not specified	Positive	Local excision	Alamri^11^/2019
Male/43 y	Distal half of the penis	Nodular lesion	Not specified	Positive	Local excision	Dunev^1^/2019
Male/45 y	Glans penis	Violaceous macule	HIV infected	Positive	Not mentioned	Asmaa^2^/2019
Male/45 y	Inner layer of prepuce	Single erythematous and slightly infiltrative macule	Not specified	Positive	Local Excision	Micali^12^/2003

The patients with KS are commonly asymptomatic but the most commonly observed complaints are pain (48.8%), edema (21.2%), bleeding (14.9), and itching (3.9%).^7^ Due to the higher possibility of non‐Hodgkin's lymphoma and malignant melanoma development in these patients, management of the lesion is very challenging. Simple excision, radiotherapy, laser, photodynamic therapy, and cryotherapy are among the recommended treatments. In addition, chemotherapy is another choice in the case of visceral involvement in these patients.^9^

## 4 CONCLUSION

Kaposi sarcoma should be considered as a differential diagnosis for erythematous nodular penile lesion. A rare presentation of KS may be present as a single lesion on the penis without any known risk factors. Therefore, histological evaluation is recommended in the patients with described penile lesions.

## CONFLICT OF INTEREST

None declared.

## AUTHOR CONTRIBUTIONS

FA: served as the main author and contributed to data acquisition and manuscript preparation. MT: contributed to data acquisition and manuscript writing. FB‐Z and ZA‐K: contributed to performing laboratory tests. RMR: served as the corresponding author and designed and supervised all the aspects and contributed to manuscript editing. All authors: contributed sufficiently and met the criteria for authorship.
